# Analysis of Dietary Pattern Impact on Weight Status for Personalised Nutrition through On-Line Advice: The Food4Me Spanish Cohort

**DOI:** 10.3390/nu7115482

**Published:** 2015-11-17

**Authors:** Rodrigo San-Cristobal, Santiago Navas-Carretero, Carlos Celis-Morales, Lorraine Brennan, Marianne Walsh, Julie A. Lovegrove, Hannelore Daniel, Wim H. M. Saris, Iwonna Traczyk, Yannis Manios, Eileen R. Gibney, Michael J. Gibney, John C. Mathers, J. Alfredo Martinez

**Affiliations:** 1 Department of Nutrition, Food Science and Physiology, Centre for Nutrition Research, University of Navarra, Pamplona 31008, Spain; rsan.1@alumni.unav.es (R.S.-C.); snavas@unav.es (S.N.-C.); 2 CIBER Fisiopatología Obesidad y Nutrición (CIBERobn), Instituto de Salud Carlos III, Madrid 28029, Spain; 3 Human Nutrition Research Centre, Institute of Cellular Medicine, Newcastle University, Campus for Ageing and Vitality, Newcastle Upon Tyne NE1 7RU, UK; carlos.celis@glasgow.ac.uk (C.C.-M.); john.mathers@newcastle.ac.uk (J.C.M.); 4 UCD Institute of Food and Health, University College Dublin, Belfield, Dublin 4, Ireland; lorraine.brennan@ucd.ie (L.B.); marianne.walsh@ucd.ie (M.W.); eileen.gibney@ucd.ie (E.R.G.); mike.gibney@ucd.ie (M.J.G.); 5 Hugh Sinclair Unit of Human Nutrition and Institute for Cardiovascular and Metabolic Research, University of Reading, Reading RG6 6AA, UK; j.a.lovegrove@reading.ac.uk; 6 Biochemistry Unit, ZIEL Research Center of Nutrition and Food Sciences, Technische Universität München, Munich 85354, Germany; hannelore.daniel@tum.de; 7 Department of Human Biology, NUTRIM, School for Nutrition and Translational Research in Metabolism, Maastricht University Medical Centre, Maastricht 6200MD, The Netherlands; w.saris@hb.unimaas.nl; 8 National Food & Nutrition Institute (IZZ), Warsaw 02-903, Poland; itraczyk@izz.waw.pl; 9 Department of Nutrition and Dietetics, Harokopio University, Athens 17671, Greece; manios@hua.gr; 10 Instituto de Investigación Sanitaria de Navarra (IdiSNA), Pamplona 31008, Spain

**Keywords:** dietary pattern, dietary habits, obesity, personalised nutrition

## Abstract

Obesity prevalence is increasing. The management of this condition requires a detailed analysis of the global risk factors in order to develop personalised advice. This study is aimed to identify current dietary patterns and habits in Spanish population interested in personalised nutrition and investigate associations with weight status. Self-reported dietary and anthropometrical data from the Spanish participants in the Food4Me study, were used in a multidimensional exploratory analysis to define specific dietary profiles. Two opposing factors were obtained according to food groups’ intake: Factor 1 characterised by a more frequent consumption of traditionally considered unhealthy foods; and Factor 2, where the consumption of “Mediterranean diet” foods was prevalent. Factor 1 showed a direct relationship with BMI (β = 0.226; *r*^2^ = 0.259; *p* < 0.001), while the association with Factor 2 was inverse (β = −0.037; *r*^2^ = 0.230; *p* = 0.348). A total of four categories were defined (Prudent, Healthy, Western, and Compensatory) through classification of the sample in higher or lower adherence to each factor and combining the possibilities. Western and Compensatory dietary patterns, which were characterized by high-density foods consumption, showed positive associations with overweight prevalence. Further analysis showed that prevention of overweight must focus on limiting the intake of known deleterious foods rather than exclusively enhance healthy products.

## 1. Introduction

Obesity, which is defined by an excessive body fat mass accumulation, is one of the most important public health problems worldwide [1]. The continuous increase in obesity prevalence has been repeatedly found to be a key factor associated with the onset of important chronic diseases [2]. Classical nutritional studies have attempted to find the relationship between the differences in the consumption of single nutritional compounds and anthropometric or biochemical markers [3]. However, the current epidemiological trends are helping to identify potential causes of obesity and accompanying comorbidities through the study of phenotypical features associated with global dietary patterns and lifestyle habits as well as the role of food exposures and their interactions [4,5,6].

Lifestyle and eating attitudes cannot be easily evaluated directly in target populations. For this reason, *a priori* dietary pattern determination (scores or indices) are used to evaluate the adherence to already known beneficial diets [7,8,9] or the adequacy/adherence to national guidelines [10], with focus on preventing metabolic disorders. On the other hand, *a posteriori* dietary pattern determinations through statistical analyses, such as principal component, factor analysis or clustering analysis, from food frequency questionnaires, allow researchers to explore the similarities of habitual food choices in specific populations and health outcomes. Subsequent post-estimation approaches can be carried out to find characteristics related to the risk of developing different non-communicable diseases as a result of long-term consumption of these patterns [11]. The study of the diversity in dietary patterns and habits also enable the inclusion of synergetic or cumulative effects of foods in association with the prevalence of obesity and its related diseases [12]. The validity of statistical determinations has been studied thoroughly in order to interpret and improve traditional dietary patterns of specific populations as well as to evaluate the biological interactions of nutrients and health [13].

The identification of dietary food patterns in large populations may contribute to identify different combinations of food choices, to assess the quality of food intake and to evaluate the effects of dietary changes for the prevention of obesity onset and the associated complications.

In this context, this research aimed to examine the lifestyle habits and dietary patterns in a Spanish cohort with interest in Personalised Nutrition (PN) by participating in the Food4Me European study, and to analyse the association with overweight and obesity prevalence. This approach tries to derive a preventative dietary pattern for obesity management and for understanding body weight regulation.

## 2. Experimental Section

### 2.1. Study Population

**Figure 1:**
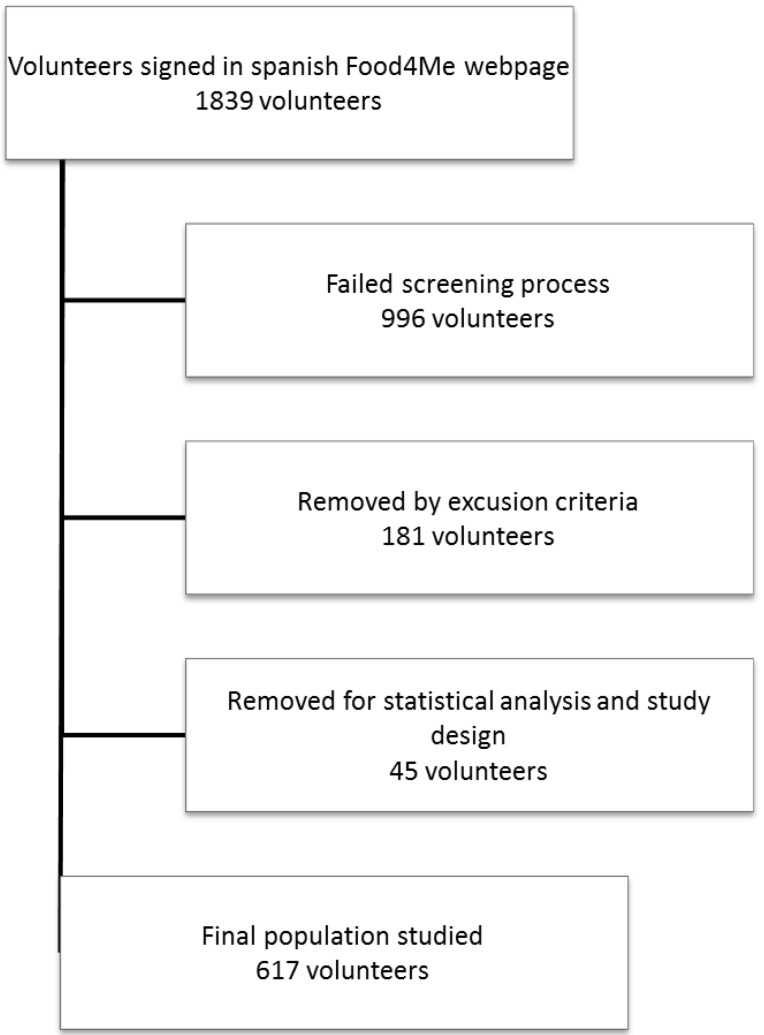
Flowchart of selection of sample.

The subjects were selected from the Food4Me project (Trial registration: NCT01530139 http://clinicaltrials.gov/show/NCT01530139). This trial was a web-based randomised controlled intervention carried out to bring about a Personalised Nutrition assessment in seven European countries [14]. All participants that signed up on the Spanish webpage (http://www.food4me.org/es/) from November 2013 to February 2014 (*n* = 1839 individuals) were selected as potential participants for the Food4Me project. To be included in the study, the volunteers had to complete two screening questionnaires, the first of them about their socio-demographic characteristics, while the second one included also medication, habits and a Food Frequency Questionnaire (FFQ). If the participants met the inclusion criteria, two consent forms were given to sign in order to proceed [14]. The criteria to be eligible to participate in the Food4Me study were the following: participants had to be more than 18 years-old, not having followed a prescribed diet within the three months prior to the study, to have access to the internet, and not to suffer any physiological condition (pregnancy) or chronic metabolic disease (Diabetes, Crohn’s disease, thyroid disorders, *etc.*). Afterwards, to ensure the validity of data collection, all the volunteers that could be considered misreporting based on the energy intake (over-reporting and under-reporting) were excluded according to the Goldberg cut-offs as updated by Black [15]. Finally the study was carried out in 617 volunteers meeting the mentioned inclusion criteria (Figure 1).

### 2.2. Dietary Assessment

The Food4Me Food Frequency Questionnaire (FFQ) was developed to assess the food and nutritional intakes of the volunteers. This survey was an online, semi-quantitative food frequency questionnaire (developed by University College Dublin and Crème Software Ltd.), based on the EPIC-Norfolk FFQ with 157 food items divided in 11 categories [16]. Validity and reproducibility of Food4Me FFQ was tested for the population studied by comparing with a four-day weighed food record [17]. To complete the FFQ the volunteers had to log-in to the website and answer the questionnaire with the average amount of the food consumed in the previous month. The volunteers received specific instructions to complete the FFQ and pictures were available to best estimate the portion size of each food item.

*A priori* dietary patterns analyses were used to compare the food intake with the adherence to healthy dietary patterns previously described. Thus, a Mediterranean-Diet Scale (MDScale) [7,11] and the Alternative Healthy Eating Index (AHEI) [18] were applied to find out similarities with the dietary patterns obtained by factor analysis. Energy intake was also reported to the basal metabolic rate ratio (EIR:BMR ratio), was calculated and analysed to avoid biases in estimation of nutrient intake [15].

### 2.3. Anthropometric Measurements

Health and anthropometric data were collected from the second screening questionnaire [14]. These data included self-reported measurements as required of the volunteers in order to select the potential participants in the study.

The validity of self-reported weight and height collected in the second screening questionnaire was examined and compared to the measurements carried out with the standardised instructions [14] by the participants. A validation study of self-reported measurements has been already carried out to ensure the reliability of data collection through the internet [19].

### 2.4. Statistical Analyses

Statistical analyses were performed using STATA statistical software (Stata IC version 12.0, StataCorp, College Station, TX, USA).

Dietary patterns were identified using factor analysis. This approach allows identification of similarities in food intakes among a large number of subjects [13], facilitating a primary classification depending on determined exposures and by reducing the number of variables introduced in the analysis. Thus, a total of 157 food items were gathered in 24 foods groups according to their nutritional value and their similar nature in order to minimize within-person variations for specific food intake (Supplementary Table S1). Subsequently, factor analysis of principal components was performed to set up the existence of one or more dietary patterns. From the statistical solution, two factors were considered to further carry out analyses taking into account the eigenvalue (greater than 2), the inflexion point on the screeplot and variance explanation of each pattern solution. Also Kaiser-Meyer-Olkin measure of sampling adequacy of overall variables was performed (greater than 0.6) to warrant the fit of factor analysis. For the factors arisen, food groups which received loading factors higher than 0.3, in absolute values, were considered as representative contributors to each dietary pattern.

In order to elucidate the relative influence of specific food groups (loading factors and absolute intakes of food) to each factor, a score was developed through a linear regression model to assess the adherence of every volunteer to both factors. A higher score indicated greater adherence to the specific pattern.

These scores were used to categorize the sample and to carry out the statistical analysis of differences in macronutrient intake, energy intake and body mass index; as well as to feature the association, through multiple linear regression, between each pattern adherence and weight status adjusting by the biological, behavioural and environmental factors. Wald tests were carried out to explore interaction effects in each level of adherence to factors. Prevalence ratio of obesity (BMI > 30 kg/m^2^) was estimated using a robust regression model. Power calculations on the analysis of differences in categories according to factors’ adherence, gave a statistical power ranging from 90% to 100% (β = 0.9093 to β = 1.000).

Subjects showing opposed adherence to each factor were categorised, in order to prevent possible deviations in the study of the resulting dietary patterns, triggered by the effects between factors.

Normal distributions were graphically verified, assuming the central limit theorem, given that the sample size was sufficiently large and the statistical distribution tests for this sample size had low power. Also homogeneity of variances, homokedasticity and absence of colinearity were checked to ensure the adequacy of the test for differences and association, respectively.

## 3. Results

### 3.1. Baseline Characteristics

The mean age of the sample was 38.0 years and 56% were females (Table 1). Significant differences in BMI and physical activity factor were found when the sample was categorised by gender and also by age (divided by the median age, 37 years). Females and younger volunteers exhibited lower BMI (24.9–27.1 and 24.7–26.9 kg/m^2^ respectively), showing females were more sedentary than males (1.48–1.52), and younger participants slightly more actives than older volunteers (1.51–1.48). Concerning macronutrient distribution, males reported higher energy, alcohol and salt intake, while females presented greater percentages in fat (total, saturated, monounsaturated and omega 3), sugar and fibre intakes. Female participants reported higher energy consumption when this was adjusted by the estimated basal metabolic rate (EIR:BMR ratio), while differences were not significant when the sample was categorised by age (1.82–1.61 and 1.74–1.73, respectively).

**Table 1:** Baseline characteristics of Spanish Food4Me volunteers included in the study.

Variable												
			Categorized by Gender					Categorized by Age				
			Female		Male		*p* ^1^	≤37 years		≤37 years		*p* ^1^
*n*	617		368		249		-	315		304		-
Age (years)	38.3 ± 9.6		37.9 ± 9.5		38.9 ± 9.8			30.7 ± 4.5		46.1 ± 7.0		-
BMI (kg/m^2^)	25.8 ± 4.5		24.9 ± 4.7		27.1 ± 3.7		***	24.7 ± 4.2		26.9 ± 4.4		***
BMI status (% of *n*)												
Normal weight	48.8%		59.8%		32.5%		*** ^3^	61.2%		36.0%		*** ^3^
Overweight	35.0%		25.5%		49.0%			29.0%		41.3%		
Obese	16.2%		14.7%		18.5%			9.9%		22.8%		
Physical activity factor	1.50 ± 4.47		1.48 ± 0.08		1.52 ± 0.11		***	1.51 ± 0.10		1.48 ± 0.09		**
Energy (kcal)	2651 ± 796		2472 ± 759		2916 ± 777		***	2632 ± 798		2670 ± 795		
EIR:BMR ratio ^2^	1.74 ± 0.50		1.82 ± 0.53		1.61 ± 0.42		***	1.74 ± 0.50		1.73 ± 0.49		
Fat (% of energy)	35.7 ± 6.4		36.3 ± 6.2		34.8 ± 6.6		**	35.7 ± 6.0		35.7 ± 6.7		
Saturated fat (% of energy)	13.1 ± 2.8		13.3 ± 2.7		12.8 ± 2.9		*	13.2 ± 2.8		13.0 ± 2.8		
Monounsaturated fat (% of energy)	14.8 ± 3.7		15.2 ± 3.8		14.3 ± 3.4		**	14.8 ± 3.5		14.9 ± 3.9		
Polyunsaturated fat (% of energy)	5.3 ± 1.3		5.4 ± 1.4		5.2 ± 1.3			5.3 ± 1.3		5.3 ± 1.4		
Omega 3 acids (% of energy)	0.82 ± 0.24		0.84 ± 0.25		0.78 ± 0.23		**	0.81 ± 0.24		0.82 ± 0.24		
Protein (% of energy)	19.2 ± 4.0		19.4 ± 4.1		19.0 ± 3.7			19.5 ± 4.0		19.0 ± 3.9		
Carbohydrate (% of energy)	44.7 ± 8.5		44.7 ± 8.4		44.7 ± 8.7			44.4 ± 7.8		45.0 ± 9.2		
Sugar (% of energy)	21.2 ± 6.8		21.9 ± 7.1		20.1 ± 6.3		**	21.2 ± 6.3		21.1 ± 7.4		
Alcohol (% of energy)	3.0 ± 3.8		2.1 ± 2.6		4.2 ± 4.8		***	2.8 ± 3.7		3.1 ± 3.9		
Salt (g)	7.7 ± 3.0		7.1 ± 2.8		8.6 ± 3.2		***	7.6 ± 3.0		7.8 ± 3.0		
Dietary fibre (g/1000 kcal)	10.6 ± 3.7		11.0 ± 3.8		10.0 ± 3.3		***	10.4 ± 3.6		10.8 ± 3.7		
Disease prevalence (% of *n*) ^4^	54.5%		57.1%		50.6%		^3^	49.0%		60.1%		** ^3^
Prescribed medication (% of *n*)	29.0%		31.8%		24.9%		^3^	23.6%		34.7%		** ^3^
Supplement user (% of *n*)	21.2%		25.3%		15.3%		** ^3^	21.0%		21.5%		^3^
Smoke (% of *n*)	16.9%		16.6%		17.3%		^3^	20.4%		13.2%		* ^3^

^1^ *p* values for *t*-test analysis: * for *p* < 0.05; ** for *p* < 0.01 *** for *p* < 0.001. ^2^ EIR:BMR, Energy Intake Reported to Basal Metabolic Rate ratio. ^3^ *p* values for chi square analisys. ^4^ Disease prevalence refers to the participant’s self-reporting in the online questionnaire of suffering from any non-communicable disease (e.g., hypertension, dyslipidaemia, osteoporosis…)

### 3.2. Factor Scores: Association and Effects with BMI

The factor analysis enabled to define two main factors (Table 2). The first one was characterised by a higher consumption of fast and processed food, potatoes, red meat, refined grains, snacks, and white meat and low intake of fruits, and vegetables. The second dietary pattern featured a higher consumption of eggs, fish products, legumes, low calorie beverages, nuts, oils, oily fruits, vegetables, and white meat.

**Table 2:** Factor loading of pattern matrix.

Variable	Factor 1	Factor 2
Alcoholic beverages		
Eggs		0.3606
Fast and processed food	0.6578	
Fat and spreads		
Fish products		0.4804
Fruits	−0.3904	
Full fat dairy products		
High fat dairy products		
Legumes		0.458
Low calorie beverages		0.3206
Nuts		0.3022
Oils		0.3305
Oily fruits		0.5014
Potatoes	0.3221	
Red meat	0.6336	
Reduced fat dairy products		
Refined grains	0.4483	
Snacks	0.6094	
Soup and sauces		
Sweets		
Sweets beverages		
Vegetables	−0.3582	0.6345
White meat	0.4622	0.3023
Whole grains		

Blanks represent absolute loading <0.3.

**Figure 2:**
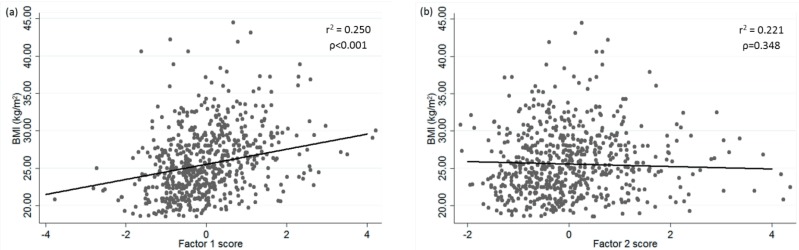
(**a**) Regression representation of BMI for Factor 1 adjusted for age, gender, energy intake, physical activity, supplement user, and smoking habit; (**b**) Regression representation of BMI for Factor 2 adjusted for age, gender, energy intake, physical activity, smoking habit, and supplement user.

Association analyses including all volunteers for each factor score showed a relationship with BMI separately and no interactions were found between factors (Figure 2a,b). The score Factor 1 showed a direct association (β = 0.226; *r*^2^ = 0.259; *p* < 0.001), while score Factor 2 presented a non-statistically significant inverse association (β = −0.037; *r*^2^ = 0.230; *p* = 0.348) with BMI when adjusted for age, gender, physical activity, smoking habits and use of supplements.

**Figure 3:**
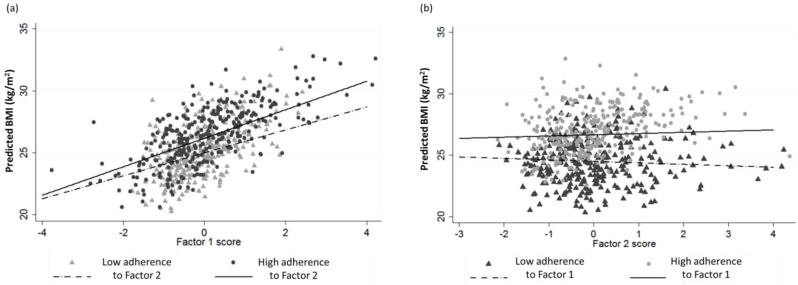
(**a**) Regression plotting of predicted BMI for Factor 1 adjusted for age, gender, energy intake, physical activity, supplement user, and smoking habit categorized by adherence to Factor 2; (**b**) Regression plotting of predicted BMI for Factor 2 adjusted for age, gender, energy intake, physical activity, smoking habit, and supplement user categorized by adherence to Factor 1.

The sample was categorized according to the adherence (scores) to each factor (Supplementary Table S2). Analyses for the differences between scores did not show statistical significant outcomes due to the wide variances, resulting in opposite scores for both factors (*i.e.*, High adherence Factor 1 + High adherence Factor 2). Consequently, analyses of interaction between high and low adherence for each factor were performed, studying the effects in the slopes for BMI relationship (Figure 3a,b). Adherence to Factor 1, presented a significant fixed effect on Factor 2 (*F* = 9.54, *p* < 0.001 for joint effect) increasing for those volunteers who had high adherence for Factor 1 (*F* = 15.89, *p* < 0.001). In the opposite analysis, slight effects were found for volunteers that presented high adherence to Factor 2 (*F* = 4.18, *p* = 0.041).

### 3.3. Dietary Patterns: Obesity Prevalence

The categorisation of volunteers depending on the adherence to Factor 1 and 2, resulted in four differentiated groups of volunteers with well-defined dietary patterns, which were denominated as “Prudent” dietary pattern for the volunteers with low adherence to both factors (*n* = 162, 126 being females), “Healthy” dietary patterns for volunteers that showed lower adherence to Factor 1 and higher adherence to Factor 2 (*n* = 147, being females 105), “Western” dietary pattern for volunteers who presented higher adherence to Factor 1 and lower adherence to Factor 2 (*n* = 147, being females 67), and “Compensatory” dietary pattern for those who had high adherence to both factors (*n* = 161, being females 70).

**Figure 4:**
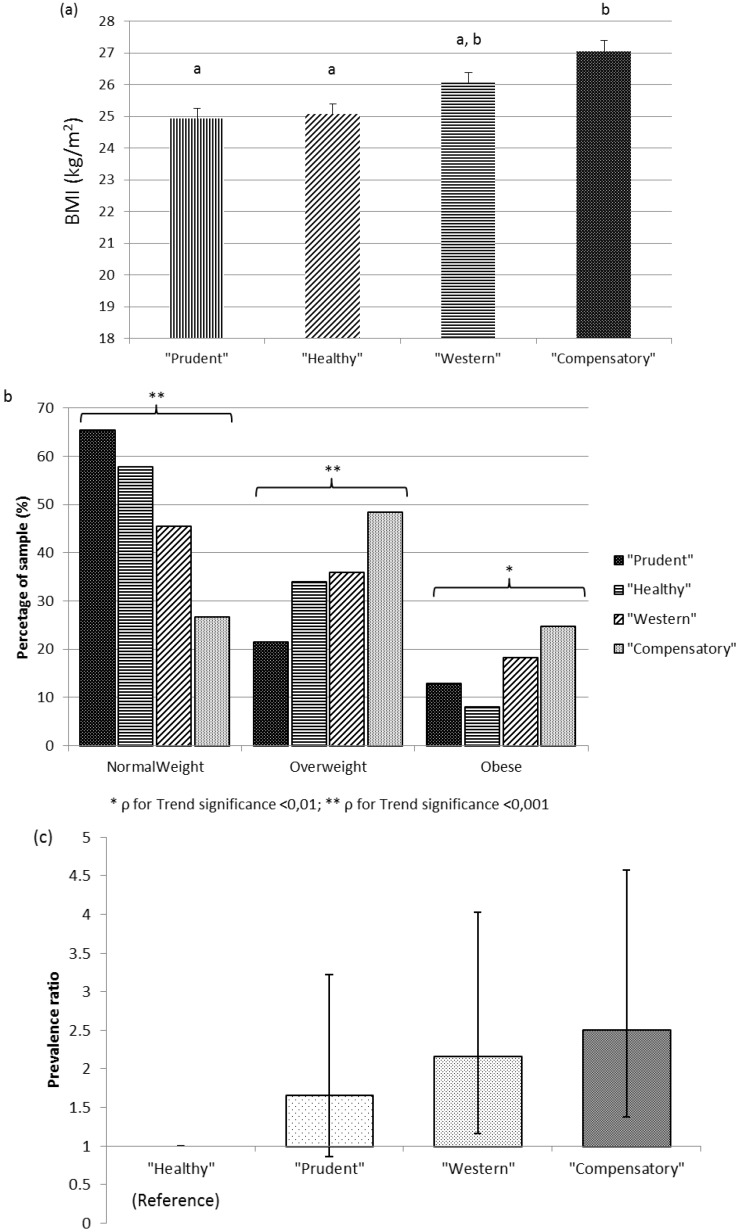
(**a**) Least square means of BMI for each dietary pattern. Values are adjusted for age, gender, energy intake reported, physical activity factor, smoking habit, and supplement user; (**b**) Prevalence of normal weight, overweight and obesity by dietary pattern; (**c**) Prevalence ratio and confidence interval (95%) for obesity (BMI ≥ 30 kg/m^2^).

The trend for prevalence of overweight (*p* < 0.001) and obesity (*p* < 0.01) increased in those dietary patterns with high adherence to Factor 1 (Western and Compensatory), while the Healthy pattern showed a reduction in obesity prevalence compared to the Prudent dietary pattern. Statistical increased odds ratio for obesity (BMI > 30 kg/m^2^) was found (Figure 4) when comparing the Healthy dietary pattern to the Western (OR = 2.66, CI: 1.22–5.81) and Compensatory (OR = 3.16, CI: 1.46–6.83) patterns, but not to the Prudent pattern (OR = 1.85, CI: 0.84–4.07).

Volunteers adhering to the “Prudent” dietary pattern showed a good fit between energy intake reported and basal metabolic rate (EIR:BMR ratio), although they did not provide any characterised dietary pattern (Supplementary Table S3), while the “Healthy” dietary pattern showed higher energy intake and an intake profile similar to Mediterranean diet (Supplementary Table S4), with a higher score on the MDScale and AHEI. Volunteers ascribed to the “Western” dietary pattern presented an energy intake reported as high as “Healthy” but with lower scores for both dietary indexes (AHEI and MDScale). Finally, volunteers that presented the “Compensatory” dietary pattern exhibited the highest energy intake, but with a slightly higher score than “Western”. According to the nutritional profile of the dietary patterns, differences for all the nutrients were observed, highlighting greatest intakes of omega 3 and dietary fibre in the Healthy dietary pattern, and highest consumption of salt in the Compensatory dietary pattern.

Differences in BMI were also observed between the dietary patterns, being “Compensatory” higher than “Prudent” and “Healthy”. Concurring to the obesity prevalence, a significantly increased obesity presence was observed among the volunteers adhering to the “Western” and “Compensatory” patterns.

### 3.4. Habits and Attitude towards Feeding

To appraise the dietary habits and attitudes of volunteers towards feeding, some questions were included in the survey. The analysis of frequencies in responses exhibited (Supplementary Figure S1) that the time preparing main meal was an important factor in the selections of recipes for the volunteers with “Western” and “Compensatory” patterns. The volunteers who reported dedicating less time to prepare the main meal and skipping meals by replacing them with snacks, were the volunteers with the “Western” pattern. These same volunteers, along with the “Compensatory” pattern subjects, presented a greater frequency in the intake of fried food, including almost one portion per week in half of the subjects of each group. Regarding the questions on how healthy was their feeding, and if this healthy eating was deliberate, “Prudent” and “Healthy” patterns exposed higher feeling to adhere to healthy habits, and these two groups reported to have those habits without having consciously to think about them.

## 4. Discussion

### 4.1. Personalised Nutrition (PN) Seekers Status

The participants were individuals interested in receiving PN through a web-based platform, and presented a similar age (40 ± 5 years) to other web-based programs for the modification of dietary and behavioural habits [20,21,22,23,24,25,26,27,28]. Concerning gender distribution, although there were wide variations in the percentage, female participants were generally found to be in higher proportion [20,21,22,23,24,25,26,29], as it occurs in the present work; only one study showed higher rates of males [28]. Concerning the BMI, the present study showed slight discrepancies in the number of overweight/obese individuals participating, compared to similar programmes [25,28] that were not specifically focused on weight-loss or directed to overweight or obese individuals. Indeed, matching our sample with the National Health Survey of 2013 [30], the weight distribution exhibited representative outcomes for Spanish population, with around 17% of obese individuals and more than half of the population exhibiting overweight or obesity [30]. Nevertheless, and despite the similarities in weight distribution, the generalization of the current outcomes to the overall Spanish population should be made with caution. The sample analysed in the present study were frequent internet users, with a specific interest on improving their health and nutritional status. The identification of a potentially interested population will be useful in order to develop further research on the predisposition for specific diseases, and prepare more tailored and targeted advice.

### 4.2. Adherence to Dietary Patterns and Obesity

The self-reported measurements used in the Food4Me study have already been validated through the assessment of the precision and the authenticity of measures [19]. In addition, the reproducibility and accuracy for the Food4Me Food Frequency Questionnaire has been also tested [16,17] as well as the study of the presence of misreporting (under and over reporting volunteers) [15], which contributes to ensure that there are no measurement errors. In addition, total energy, smoking, supplements consumption and physical activity level adjustments have been used to reduce inter-individual variance and to control for confounding factors in the prediction of dietary intake [31,32].

The multidimensional exploratory analysis from a matrix of foods sheds light on synergistic and additive effects of nutrients contained in food [4]. However, the reproducibility of this type of analyses may turn into a too laborious work, taking into account the variety of foods in the different regions worldwide [13]. Nevertheless, dietary patterns seem to be stable across demographical variances in other studied populations [33], and dietary patterns based on foods or food groups consumption probably have an easier interpretation and thus implementation in nutritional assessment [34].

The number of dietary patterns obtained from other studies varied from two to eight [35], and food-groups or food items included in each pattern differed [35,36]. However, it has been shown that there is a direct association between food content within the different dietary patterns, and the increase of body weight. Thus, Sun *et al*. [37] established that adults with a Western dietary pattern have a higher waist-hip ratio, BMI and relative risk of obesity hypertension, metabolic syndrome, and dyslipidaemia in adults. Also, Newby *et al*. [38] found in a prospective study that the individuals following a dietary pattern characterised by greater energy contributions from fruits, high-fibre cereal, and reduced fat dairy, with smaller contributions from fast food, non-diet soda, and salty snacks were associated with smaller gains in BMI and waist circumference. In this context, Flores *et al*. [39] showed an increase in the odds ratio to be obese in subjects that had higher energy intake from alcohol, soft drinks, white bread, fast food, sweets and candies, and salty snacks, and lowest contribution from maize and the highest proportion of whole-fat dairy, rice and pasta, meat, poultry, eggs, saturated fat, fruits, and vegetables, compared to a traditional dietary pattern with main intake resulting from maize foods.

There are also other studies that have established a connection between dietary patterns with overlapping food groups and the variance on body composition in children and adolescents [40,41], changes in the risk of metabolic syndrome [42,43,44] or other relevant chronic diseases [45,46]. Therefore, the evidence found in the present study contributes to confirm these previous findings.

Additional epidemiological studies suggest that the current Spanish dietary pattern is in transition [47], as it has been observed in other Mediterranean and European countries [48], from a traditional Mediterranean lifestyle to imported Western habits [49]. Migrations, socioeconomical factors and larger variety of food availability promotes the evolution of traditional dietary patterns [50], and seems to cause “Westernization”.

In this context, the Compensatory dietary pattern, established in the current research, may be identified in those volunteers who have adopted western dietary habits, but try to “*compensate*” this unhealthy pattern by increasing the consumption of “*healthy food*” or “*healthy snacks*” in addition to current intake [51,52,53,54]. This attempt of improvement leads to an overconsumption of energy with the subsequent weight gain [55,56]. These findings could help to explain the existing hypothesis of Mediterranean paradox [57].

Following this concept, the study of the Mediterranean paradox may be studied through the statistical analysis of dietary habits proposed in this study, which would allow the identification of the current clusters of diets and their evolution, preventing potential confounders [58], and not limiting the classification of individuals to a specific adherence.

In addition to dietary patterns, the study of attitudes towards diet and food may be considered an important tool for profiling individuals, and estimating the most suitable advice to obtain the most effective response in weight loss programs. Variables such as the advice provider (whose profession is delivering dietary advice), or concerns on how the information provided is held [59,60] can affect the acceptance of personalised nutrition advice.

However, it is important to take into account other synergistic conditions for overweight and obesity, such as genetic factors [61]. Although diet and lifestyle are triggers to maintain or increase body weight and fat mass, the success of prescribed dietary programs may also rely on the presence of determined SNPs related with the metabolism of different nutrients [62]. In addition, those dietary habits may modulate epigenetic marks in earlier life-stages that could alter the disease risk and may also modify the response to futures dietary interventions [63].

### 4.3. Tailoring the Advice Based on Prediction of Dietary Behaviours

Based on the results obtained in the current analyses, and taking into account the previously discussed Mediterranean paradox [57], it seems of major importance to enhance Public Health Advice with the use of feasible and relatively simple tools to diagnose and predict specific targets for specific dietary patterns [64].

If a patient shows a “Compensatory” eating behaviour, the advice on increasing omega-3, shall be led to a secondary scenario, and the reduction of Factor 1 foods may be more effective to prevent future diseases. Furthermore, overconsumption of energy, with an important component of oils and snacks, may be a major contributor of this inflammation, which would revert more rapidly than increasing omega-3 intake. Nevertheless, sensible and evidence-based nutritional advice should always prevail [61]; it may be proposed to further explore changes in the priority of the advice, depending on subjects’ behaviours.

## 5. Conclusions

The results obtained in the present study suggest that statistical analyses of dietary intake from populations help to describe current dietary patterns, which facilitate the targeting of nutritional objectives to tailor dietary management. Furthermore, prevention of overweight may not be reached by only encouraging the inclusion of healthy choices, but also by a specific stress on limiting deleterious foods.

## Supplementary Materials

Supplementary materials can be accessed at: http://www.mdpi.com/2072-6643/7/11/5482/s1.
