# Supplementary material for: Analysis of Dietary Pattern Impact on Weight Status for Personalised Nutrition through On-Line Advice: The Food4Me Spanish Cohort
Source: Nutrients. 2015 Nov 17;7(11):9523–37. doi: 10.3390/nu7115482 (PMC4663610; doi:10.3390/nu7115482)
Supplement: Supplementary file 1 [file nutrients-07-05482-s001.docx]

**Supplementary Materials: Analysis of Dietary Pattern Impact on Weight Status for Personalised Nutrition through On-Line Advice: The Food4Me Spanish Cohort**

Rodrigo San-Cristobal, Santiago Navas-Carretero, Carlos Celis-Morales, Lorraine Brennan, Marianne Walsh, Julie A. Lovegrove, Hannelore Daniel, Wim H. M. Saris, Iwonna Traczyk, Yannis Manios, Eileen R. Gibney, Michael J. Gibney, John C. Mathers and J. Alfredo Martinez

2. Experimental Section

2.4. Statistical Analyses

**Table S1.** Description of food groups.

| **Food Group** | **Foods** |
| --- | --- |
| **Alcoholic beverages** | Beer, larger, cider, sweet alcoholic drinks, cocktails, wine, spirits, brandy, whiskey, vodka; Port, sherry, vermouth, liqueurs |
| **Egg** | Boiled, scrambled, omelette |
| **Fast & processed food** | Lasagne, moussaka, ravioli and tortellini, filled dumplings, fish fingers, fish cakes, spring rolls, fried fish in batter, burgers, meatballs, processed chicken or poultry, goujons, pizza, calzone, savoury pies, meat pies, pasties, sausage rolls, quiche, savoury pancakes |
| **Fat and spreads** | Polyunsaturated marg flora/sunflower/soya, butter |
| **Fish products** | Shellfish, crab, prawn, fish dishes, white fish, haddock, sole, sushi, fresh non-smoked oily fish, canned non-smoked oily fish, fish roe, caviar, taramasalata, smoked fish |
| **Fruits** | Grapefruit, berries, oranges, satsumas, mandarins, peaches, plums, apricots, pure fruit juice, pears, melon, mango, apples, kiwi, grapes, bananas, tinned stewed fruit, dried fruit |
| **Full fat dairy products** | Full fat whole milk average, milkshakes, fruit smoothies, milk puddings, hot chocolate, Ovaltin or, Horlicks made with milk, full fat  Greek yoghurt |
| **High fat dairy products** | Medium fat cheeses: edam, goats, camembert, feta, emmental; Low fat cheeses: fresh mozzarella, cream cheese; high fat cheeses: stilton, cheddar, brie, gouda; single/sour cream; soluble/clotted cream |
| **Legumes** | Baked beans, fresh/frozen peas, tofu, dried lentils, beans, peas, chickpeas |
| **Low calorie beverages** | Coffee (americano, black), tea (black, green, fruit, herbal), low calorie/ diet fizzy soft drinks |
| **Nuts and seeds** | Almonds, peanuts, pumpkin seeds |
| **Oils** | Other vegetable oils, olive oil |
| **Oily Fruits** | Olives, avocado |
| **Potatoes** | Boiled, jacket, mashed, instant, roast, potato dishes (salads, dauphinoise) |
| **Red meat** | Stew and casserole (meat and veg); offal, lamb, goat, beef, venison, cured meats, sausages, bacon, pate |
| **Reduced fat dairy products** | Coffee (milky, latte, cappuccino), zero fat/skimmed milk, low-fat/semi-skimmed milk, low-fat natural yoghurt, fruit yoghurt, fruit mousse, very low fat cheese |

Table S1. *Cont.*

| **Food Group** | **Foods** |
| --- | --- |
| **Soup and sauces** | Non-creamy soups, low fat salad cream, mayonnaise, salad cream, dark sauces, creamy soups, tomato ketchup; tomato sauces, marmite/Bovril, creamy sauces, other salad dressing, French dressing, vinaigrette |
| **Snacks** | Pickles, chutney, satesaus (sate), chips, cream crackers, cheese biscuits, rusks, crisps or other packet snacks |
| **Sweets** | Ice-cream, choc ices, jam, marmalade, honey, fruit pies, tarts, crumbles, sweets, toffees, mints, liquorice, sugar added to tea or coffee, rich cakes, sweet biscuits, chocolate snack bars, chocolates, nut or chocolate spreads |
| **Sweets beverages** | Hot chocolate, Ovaltine or Horlicks made with water, sorbets and jellies, fruit squash/cordial/nectar, fizzy soft drinks |
| **Vegetables** | Green salad, lettuce, cucumber, celery, watercress, cabbage, tomato and vegetable juices, asparagus, okra, leeks; spinach, sweet peppers, cauliflower, broccoli, spring greens, kale, carrots, parsnips, turnips, swedes, butternut squash, pumpkin, Brussels sprouts, beetroot, tomatoes, beans sprouts, radish, green beans, broad beans, runner beans, onions, corn (on the cob, sweet corn), garlic, marrow, courgettes, aubergine, mushrooms, coleslaw, sauerkraut |
| **White meat** | Sliced cold meats, chicken or poultry, pork |
| **Whole Grains** | Wholemeal pasta, brown rice, dark wholemeal breads, brown and seeded bread, brown and seeded rolls, wholegrain breakfast cereals, muesli, flapjacks, muesli bars, oatmeal cookies |

3. Results

3.2. Factor Scores: Association and Effects with BMI

**Table S2.** Score means, ρ values and coefficients (β) for regression model for BMI, categorised by adherence to each factor.

|  | **Overall** | | **Adherence to Factor 1** | | | | | **Adherence to Factor 2** | | | | |
| --- | --- | --- | --- | --- | --- | --- | --- | --- | --- | --- | --- | --- |
|  |  |  | **Low** | | **High** | | ***p* ^1^** | **Low** | | **High** | | ***p* ^1^** |
| *n* | 617 | | 309 | | 308 | | - | 309 | | 308 | | - |
| Score for Factor 1 | 0.00 ± 1.00 | | −0.76 ± 0.51 | | 0.76 ± 0.77 | | - | −0.08 ± 0.79 | | 0.08 ± 1.17 | |  |
| Score for Factor 2 | 0.00 ± 1.00 | | 0.00 ± 1.02 | | 0.00 ± 0.98 | |  | −0.75 ± 0.42 | | 0.75 ± 0.84 | | - |
|  | β | *p* ^2^ | β | *p* ^2^ | β | *p* ^2^ |  | β | *p* ^2^ | β | *p* ^2^ |  |
| **Factor 1** |  |  |  |  |  |  |  |  |  |  |  |  |
| Adjusted for age and gender | 0.326 | <0.001 | 0.165 | 0.002 | 0.207 | <0.001 |  | 0.267 | <0.001 | 0.366 | <0.001 |  |
| Multivariate model ^3^ | 0.224 | <0.001 | 0.139 | 0.007 | 0.114 | 0.091 |  | 0.170 | 0.009 | 0.291 | <0.001 |  |
| **Factor 2** |  |  |  |  |  |  |  |  |  |  |  |  |
| Adjusted for age and gender | 0.032 | 0.404 | −0.012 | 0.831 | 0.088 | 0.108 |  | −0.019 | 0.719 | −0.092 | 0.094 |  |
| Multivariate model ^3^ | −0.038 | 0.337 | −0.034 | 0.566 | 0.022 | 0.716 |  | −0.007 | 0.897 | −0.138 | 0.009 |  |

^1^ *p*-value for *t*-test. ^2^ *p*-value for variable in regression model. ^3^ Multivariate model adjusted for age, gender, physical activity, smoking and suplement user.

3.3. Dietary Patterns: Obesity Prevalence

**Table S3.** Characteristics of sample categorised by dietary patterns.

| **Variable** | **Prudent** | | **Healthy** | | **Western** | | **Compensatory** | | ***p* ^1,2^** |
| --- | --- | --- | --- | --- | --- | --- | --- | --- | --- |
| *n* (*n* of females) | 162 (126) |  | 147 (105) |  | 147 (67) |  | 161 (70) |  |  |
| Age (years) | 38.8 ± 10.3 | ^a^ | 39.6 ± 9.7 | ^a^ | 36.2 ± 9.5 | ^b^ | 38.5 ± 8.7 | ^a,b^ | ** |
| BMI (kg/m²) | 24.3 ± 4.0 | ^a^ | 24.7 ± 4.1 | ^a^ | 26.2 ± 4.4 | ^a,b^ | 27.9 ± 4.4 | ^b^ | ** |
| Score for Factor 1 | −0.68 ± 0.37 | ^b^ | −0.85 ± 0.61 | ^a^ | 0.58 ± 0.57 | ^c^ | 0.93 ± 0.88 | ^c^ | *** |
| Score for Factor 2 | −0.72 ± 0.43 | ^b^ | 0.79 ± 0.89 | ^d^ | −0.78 ± 0.42 | ^a^ | 0.71 ± 0.78 | ^c^ | *** |
| Alternative Healthy Eating Index | 57.10 ± 8.85 | ^c^ | 63.26 ± 9.83 | ^d^ | 47.31 ± 8.77 | ^a^ | 50.89 ± 9.68 | ^b^ | *** |
| Mediterranean diet scale | 4.91 ± 1.77 | ^b^ | 6.03 ± 1.57 | ^c^ | 3.70 ± 1.76 | ^a^ | 5.02 ± 1.73 | ^c^ | *** |
| Physical activity factor | 1.50 ± 0.09 | ^b^ | 1.51 ± 0.09 | ^b^ | 1.48 ± 0.10 | ^a^ | 1.50 ± 0.11 | ^a,b^ | ** |
| EIR:BMR ratio ^3^ | 1.49 ± 0.40 | ^a^ | 1.76 ± 0.45 | ^b^ | 1.66 ± 0.47 | ^b^ | 2.02 ± 0.50 | ^c^ | *** |
| Energy (kcal) | 2087 ± 522 | ^a^ | 2514 ± 606 | ^b^ | 2655 ± 753 | ^b^ | 3339 ± 706 | ^c^ | *** |
| Fat (% of energy) | 34.5 ± 6.2 | ^a^ | 36.4 ± 6.6 | ^b,c^ | 34.8 ± 6.6 | ^a,b^ | 37.0 ± 5.8 | ^c^ | *** |
| Saturated fat (% of energy) | 13.0 ± 2.9 | ^a,b^ | 12.3 ± 2.5 | ^a^ | 13.5 ± 2.9 | ^b^ | 13.5 ± 2.6 | ^b^ | *** |
| Monounsaturated fat (% of energy) | 14.1 ± 3.6 | ^a^ | 15.8 ± 4.2 | ^b^ | 13.9 ± 3.2 | ^a^ | 15.5 ± 3.3 | ^b^ | *** |
| Polyunsaturated fat (% of energy) | 4.9 ± 1.1 | ^a^ | 5.8 ± 1.6 | ^b^ | 5.0 ± 1.3 | ^a^ | 5.5 ± 1.1 | ^b^ | *** |
| Omega 3 acids (% of energy) | 0.82 ± 0.26 | ^b^ | 0.93 ± 0.24 | ^c^ | 0.70 ± 0.18 | ^a^ | 0.82 ± 0.22 | ^b^ | *** |
| Protein (% of energy) | 18.7 ± 4.4 | ^a^ | 19.5 ± 3.6 | ^a,b^ | 18.6 ± 4.1 | ^a^ | 20.1 ± 3.6 | ^b^ | ** |
| Carbohydrate (% of energy) | 47.4 ± 7.6 | ^b^ | 43.5 ± 8.7 | ^a^ | 46.2 ± 8.7 | ^b^ | 41.7 ± 8.0 | ^a^ | *** |
| Sugar (% of energy) | 24.4 ± 7.1 | ^d^ | 22.6 ± 7.0 | ^c^ | 20.0 ± 6.1 | ^b^ | 17.7 ± 5.1 | ^a^ | *** |
| Sugar without fruit (%of energy) | 16.2 ± 5.0 | ^b^ | 13.2 ± 4.4 | ^a^ | 16.2 ± 5.7 | ^b^ | 13.3 ± 4.1 | ^a^ | *** |
| Alcohol (% of energy) | 2.5 ± 3.2 |  | 2.6 ± 2.8 |  | 3.3 ± 4.8 |  | 3.4 ± 4.1 |  |  |
| Salt (g) | 5.6 ± 1.6 | ^a^ | 7.1 ± 2.3 | ^b^ | 7.9 ± 2.9 | ^b^ | 10.2 ± 3.0 | ^c^ | *** |
| Dietary fibre (g/1000 kcal) | 11.3 ± 3.3 | ^c^ | 13.7 ± 3.8 | ^d^ | 8.1 ± 2.2 | ^a^ | 9.3 ± 2.5 | ^b^ | *** |
| Disease prevalence (% of n) | 53.1% |  | 59.9% |  | 51.7% |  | 53.4% |  | ^4^ |
| Smoke (% of n) | 16.1% |  | 10.9% |  | 21.8% |  | 18.6% |  | ^4^ |

^1^ *p* values for ANOVA analysis adjusted by Age, Gender, Physical Activity Factor and Energy intake: * for *p* < 0.05; ** for *p* < 0.01 *** for *p* < 0.001 and superscript letters show differences (*p*<0.05) when letters are different. ^2^ *p* values adjusted by multiple-test procedure Benjamini Hochberg. ^2^ Energy Intake Reported: Basal Metabolic Rate ratio. ^3^ *p* values for chi square analysis.

3.4. Habits and Attitude towards Feeding

**Table S4.** Food composition of dietary patterns in grams per 1000 kcal.

| **Variable** | **Prudent** | | **Healthy** | | **Western** | | **Compensatory** | | ***p* ^1,2^** |
| --- | --- | --- | --- | --- | --- | --- | --- | --- | --- |
| Fruits | 210.2 ± 139.6  81.0 ± 49.1  25.1 ± 28.7  18.1 ± 12.9 | ^b^ | 245.0 ± 164.0  60.7 ± 39.9  36.1 ± 37.9  21.3 ± 14.6  29.6 ± 19.9  44.4 ± 31.7  156.6 ± 112.0  30.6 ± 52.7  10.8 ± 11.0  0.8 ± 1.6  7.6 ± 6.3  3.0 ± 5.4  52.6 ± 54.5  20.0 ± 17.3  16.1 ± 12.0  13.5 ± 11.8  10.4 ± 16.9  126.2 ± 79.9  131.0 ± 147.0  18.5 ± 12.4  5.9 ± 4.6  25.7 ± 43.3  42.4 ± 40.3  12.2 ± 12.0 | ^b^ | 94.2 ± 73.7  105.5 ± 65.2  9.6 ± 18.4  32.4 ± 21.6  30.5 ± 20.3  21.3 ± 13.9  190.0 ± 152.5  41.9 ± 65.1  6.4 ± 5.9  1.0 ± 1.5  3.8 ± 3.6  1.1 ± 1.9  76.1 ± 126.7  11.1 ± 9.7  12.9 ± 9.3  14.2 ± 13.5  1.8 ± 2.4  36.4 ± 21.5  54.5 ± 82.8  34.7 ± 19.9  17.0 ± 12.6  54.1 ± 74.8  32.3 ± 23.9  19.2 ± 19.3 | ^a^ | 112.5 ± 80.9  89.9 ± 44.7  16.1 ± 21.8  34.0 ± 21.3  37.1 ± 19.2  28.8 ± 18.9  162.3 ± 131.4  25.0 ± 36.1  9.2 ± 8.9  1.2 ± 2.7  5.9 ± 4.4  1.5 ± 2.4  74.4 ± 90.0  16.1 ± 13.1  16.8 ± 13.0  17.7 ± 14.1  3.4 ± 5.2  58.6 ± 34.4  98.7 ± 115.6  33.6 ± 19.5  11.8 ± 8.8  34.6 ± 58.1  37.0 ± 28.8  14.9 ± 11.3 | ^a^ | *** |
| Refined grains |  | ^b^ |  | ^a^ |  | ^c^ |  | ^b^ | *** |
| Whole grains |  | ^b^ |  | ^c^ |  | ^a^ |  | ^a^ | *** |
| Red meat |  | ^a^ |  | ^a^ |  | ^b^ |  | ^b^ | *** |
| White meat | 28.6 ± 18.9  33.3 ± 24.0  230.5 ± 169.9  38.7 ± 60.4  8.3 ± 8.5  0.7 ± 1.3  5.7 ± 5.1  1.4 ± 2.4  55.2 ± 70.0  12.9 ± 12.3  12.9 ± 11.7  10.7 ± 11.5  2.9 ± 4.5  75.0 ± 40.4  82.9 ± 106.8  20.3 ± 13.7  7.6 ± 7.4  29.7 ± 62.7  30.7 ± 28.7  21.4 ± 22.0 | ^a^ |  | ^a^ |  | ^a^ |  | ^b^ | ** |
| Fish products |  | ^b^ |  | ^c^ |  | ^a^ |  | ^b^ | *** |
| Reduced fat dairy products |  | ^c^ |  | ^a^ |  | ^b,c^ |  | ^a, b^ | *** |
| Full fat dairy products |  |  |  |  |  |  |  |  |  |
| High fat dairy products |  | ^a,b^ |  | ^c^ |  | ^a^ |  | ^b, c^ | ** |
| Fat and spreads |  |  |  |  |  |  |  |  |  |
| Oils |  | ^b^ |  | ^c^ |  | ^a^ |  | ^b, c^ | *** |
| Nuts |  | ^a^ |  | ^b^ |  | ^a^ |  | ^a^ | *** |
| Alcoholic beverages |  |  |  |  |  |  |  |  |  |
| Legumes |  | ^a,b^ |  | ^c^ |  | ^a^ |  | ^b,c^ | *** |
| Eggs |  | ^a^ |  | ^a,b^ |  | ^a^ |  | ^b^ | * |
| Potatoes |  | ^a^ |  | ^a,b^ |  | ^b,c^ |  | ^c^ | *** |
| Oily fruits |  | ^a^ |  | ^b^ |  | ^a^ |  | ^a^ | *** |
| Vegetables |  | ^b^ |  | ^c^ |  | ^a^ |  | ^b^ | *** |
| Low calorie beverages |  | ^a,b^ |  | ^c^ |  | ^a^ |  | ^b,c^ | *** |
| FastFood |  | ^a^ |  | ^a^ |  | ^b^ |  | ^b^ | *** |
| Snacks |  | ^a^ |  | ^a^ |  | ^c^ |  | ^b^ | *** |
| Sweets beverages |  | ^a^ |  | ^a^ |  | ^b^ |  | ^a^ | ** |
| Soup and sauces |  | ^a^ |  | ^b^ |  | ^a,b^ |  | ^a,b^ | * |
| Sweets |  | ^c^ |  | ^a^ |  | ^b,c^ |  | ^a,b^ | *** |

^1^ *p* values for ANOVA analysis adjusted by Age and Gender: * for *p* < 0.05; ** for *p* < 0.01 *** for *p* < 0.001 and superscript letters show differences (*p*<0.05) when letters are different. ^2^ *p* values adjusted by multiple-test procedure Benjamini Hochberg.


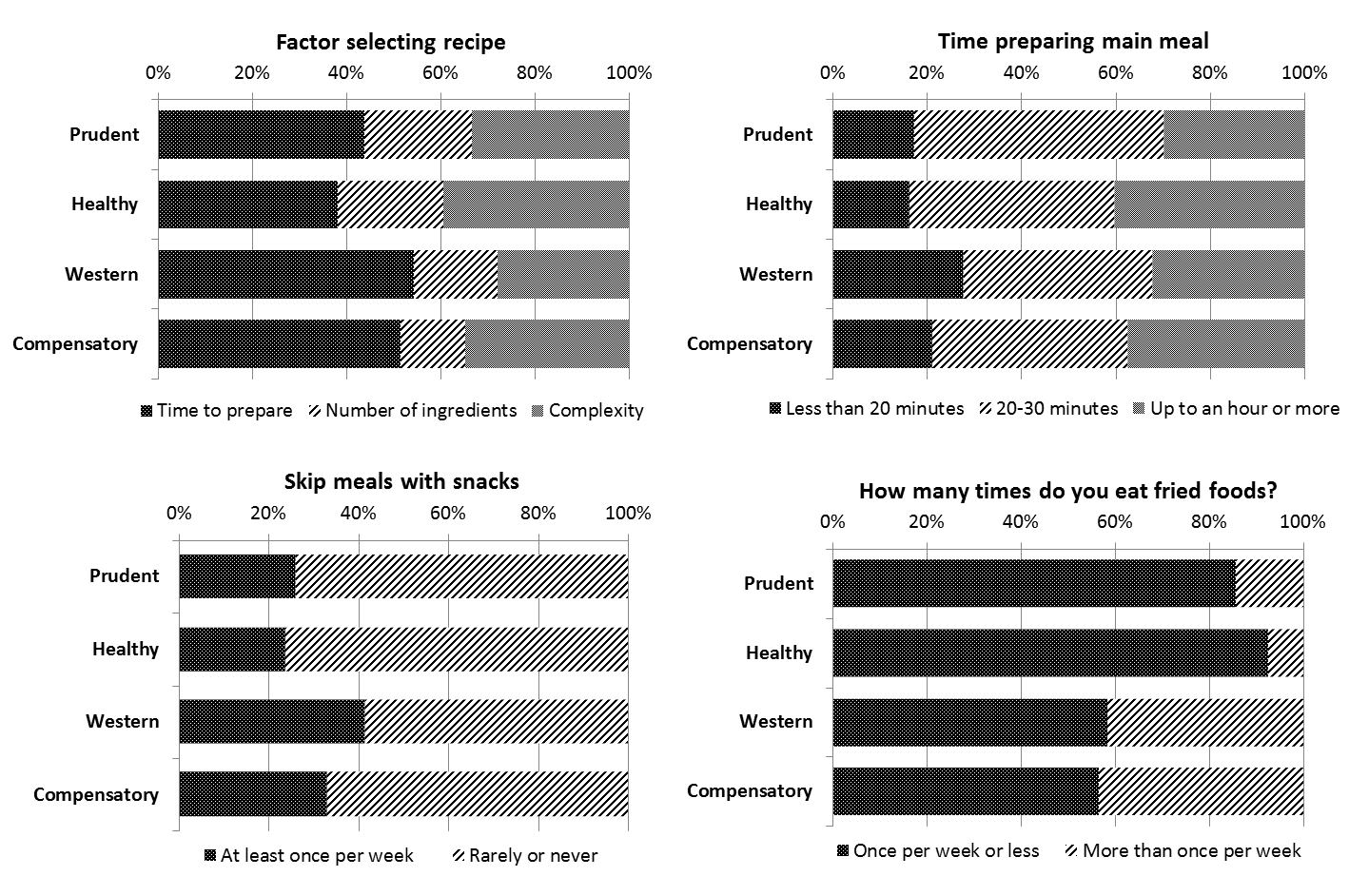


**Figure S1.** *Cont.*


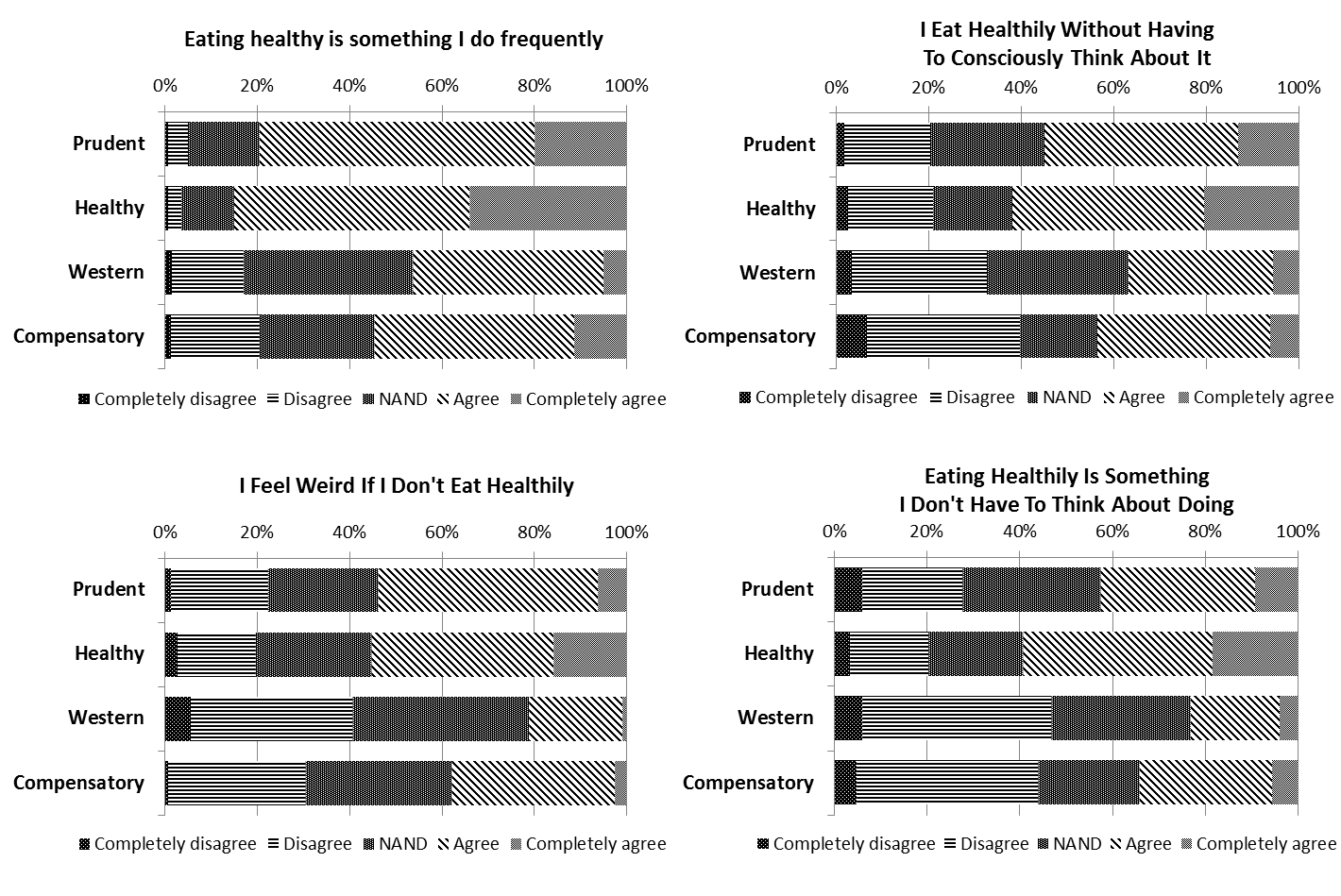


**Figure S1.** Dietary habits to each dietary pattern and healthy eating feeling to each dietary pattern.
